# The Jejunojejunostomy: an Achilles Heel of the Roux-en-Y Gastric Bypass Construction

**DOI:** 10.1007/s11695-021-05686-2

**Published:** 2021-09-03

**Authors:** Suzanne Hedberg, Yao Xiao, Adam Klasson, Almantas Maleckas, Mikael Wirén, Anders Thorell, Anna Laurenius, My Engström, Torsten Olbers

**Affiliations:** 1 Department of Surgery, Institute of Clinical Science, Sahlgrenska Academy, University of Gothenburg, Diagnosvägen 11, 416 50 Gothenburg, Sweden; 2 Department of Surgery, Region Västra Götaland, Sahlgrenska University Hospital, Gothenburg, Sweden; 3 Department of Surgery, Medical Academy, Lithuanian University of Health Sciences, Kaunas, Lithuania; 4 Department of Clinical Sciences, Danderyd Hospital, Karolinska Institute, Stockholm, Sweden; 5 Department of Surgery, Ersta Hospital, Stockholm, Sweden; 6 Institute of Health and Care Sciences, Sahlgrenska Academy, University of Gothenburg, Gothenburg, Sweden; 7 Department of Biomedical and Clinical Sciences, University of Linköping, Norrköping, Sweden

**Keywords:** Obesity, Bariatric Surgery, Roux-en-Y gastric bypass, Revision, Obstruction

## Abstract

**Background:**

Laparoscopic Roux-en-Y gastric bypass (RYGB) has for long been the gold standard technique in bariatric surgery, especially in the Scandinavian countries. In a tertiary hospital setting, we observed an increasing number of patients with postprandial abdominal pain and nausea, often associated with complex hypoglycemia.

**Objectives:**

The present study aimed to characterize the clinical patterns, patient characteristics, and clinical outcomes after surgical revision of dysfunctional RYGB at Sahlgrenska University Hospital in Gothenburg, Sweden.

**Methods:**

This cohort study included patients with RYGB who underwent revision of the jejunojejunostomy (JJ) after 2013. Information was obtained by reviewing medical records and performing complementary interviews.

**Results:**

Laparoscopic revisional surgery was performed in 115 cases with either adhesiolysis or total revision of the JJ (mean age 41 years, range 19–67 years; 90% women). The median time to assessment after the last revision was 33 months (range 12–75 months). Forty-four (38%) patients reported that they were symptom-free long-term after the intervention, and 32 (28%) patients experienced an improvement in the symptoms that were the indication for revision. However, 31 (27%) patients reported no long-term improvement, and half of them (*n* = 16) subsequently had a reversal of the anatomy. Eight (7%) patients were lost to follow-up.

**Conclusions:**

Dysfunction of the JJ appears to be a relatively common cause of postprandial pain and nausea after ante-colic/ante-gastric RYGB. Most patients with symptoms of dysfunction experienced partial or total relief following revisional surgery, but a substantial minority had persistent problems, with one in five eventually undergoing reversal of the anatomy.

**Graphical Abstract:**

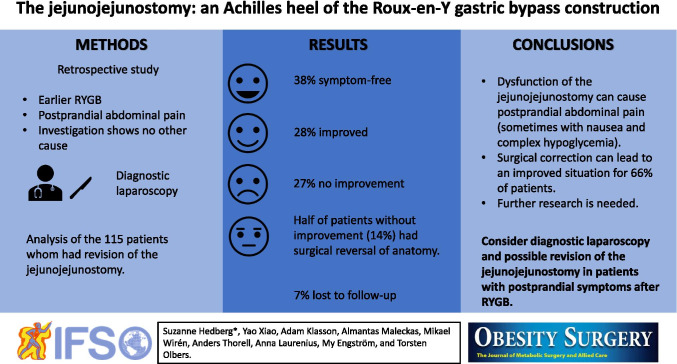

## Introduction

Laparoscopic Roux-en-Y gastric bypass (RYGB) has since long been the gold standard in bariatric surgery and in Scandinavia performed according to the Lönroth method for more than two decades [1]. Approximately half of all bariatric surgeries in Sweden are currently RYGBs, almost always by laparoscopy, with a conversion rate of < 1% [2]. Sahlgrenska University Hospital is a tertiary referral center for bariatric surgery in a region with approximately 2 million inhabitants. Patients suffering from complications following bariatric surgery, both early and late, who cannot be managed at local centers, are referred to this tertiary center. We have observed an increased number of referrals due to complications since 2012.

Studies suggest that as many as 22–30% of patients who undergo RYGB experience postoperative abdominal pain [3, 4]. Patients with abdominal pain after RYGB are normally investigated for gall stones, ulcers at the gastrojejunostomy, dumping, hypoglycemia, and other well-known complications. In our practice, all patients with complications were seen by dieticians with specialist knowledge of bariatric surgery patients to handle dumping and simple hypoglycemic problems, in addition to investigations by the surgeons. However, for an increasing number of patients, we have not found a “traditional” diagnosis, and a substantial number of patients with abdominal pain, especially postprandial abdominal pain, have emerged. In addition to pain, most often in the upper left quadrant, there are frequent complaints of postprandial nausea and in many cases also postprandial hypoglycemia [5].

The increasing number of symptomatic patients coincides with an overall increase in the number of RYGB procedures performed in Sweden, but also with the introduction of the closure of mesenteric defects. A randomized trial in Sweden demonstrated a 3-year benefit of mesenteric window closure, but also an increased need for remedial surgery due to small bowel obstruction during the first year after surgery, in patients with mesenteric defect closure [6].

Patients with abdominal pain after RYGB often undergo one or several abdominal computer tomography (CT) scans. However, studies have shown that a negative CT scan does not necessarily exclude pathology that could benefit from surgical correction [7–10]. Subtle findings are often overlooked, such as the abnormal location of the jejunojejunostomy (JJ), or retrograde passage of contrast into the bilio-pancreatic limb, duodenum, and even the gastric remnant. Upper gastrointestinal follow through X-ray, especially with a food challenge, may be more sensitive for demonstrating partial small bowel obstructions, but the method has not been standardized or scientifically evaluated. Therefore, a diagnostic laparoscopy is usually recommended in patients with suggestive symptoms in the absence of significant radiological findings [7, 10].

A clinical observation emerged that adhesions and kinking at the JJ appear to lead to partial obstruction as suggested by the presence of postprandial pain in the upper left quadrat often in conjunction with nausea and complex hypoglycemia. This is in corroboration with data from a few case reports and smaller case series that addressed small bowel obstruction after bariatric surgery [11–14]. In addition to internal herniation, adhesion, intussusception, and blood clots at the JJ have also been demonstrated. To the best of our knowledge, no study has previously defined and systematically characterized such surgical problems at the JJ and subsequent outcomes after intervention.

This study retrospectively characterized symptoms suggestive of significant dysfunction at the JJ and, in a cross-sectional assessment, investigated the outcomes of patients who underwent revisional surgery.

## Materials and Methods

We identified patients with RYGB who underwent revisional surgery at Sahlgrenska University Hospital with a suspicion of dysfunction at the JJ from January 2012 to February 2017 using the registered codes from the Nordic Medico-Statistical Committee (NOMESCO) Classification of Surgical Procedures (JAW97, JFB00, JAP01, JFC01, JFK97). Accurate coding was verified using the medical charts. The patient’s medical history before and after the revision was retrieved from medical records using a predefined list of variables, including preoperative demographics, surgical information from the primary RYGB, onset of symptoms, and type of surgical correction in subsequent remedial surgery. Patients were contacted for a complementary semi-structured telephone interview to identify any current and previous complaints that could not be retrieved from the charts, such as pain and symptoms suggestive of hypoglycemia. In addition, patient-reported body mass index (BMI) and an overall assessment of symptom relief after revisional surgery were obtained.

The primary endpoint was the effectiveness of the surgical intervention in alleviating the symptoms that were perceived as being associated with JJ dysfunction. At the time of the study follow-up, patients were classified as symptom-free, improved, or unchanged/persistent symptoms. The classification was based on a combination of the patient’s experience and symptoms documented in their medical record.

Secondary endpoints were demographic factors that possibly added risk for surgical complications (i.e., previous abdominal surgery, smoking, diabetes) and individual factors that might make coping with complications more complex (i.e., psychological/psychiatric diseases, previous chronic pain syndrome). The number of surgeries the patients had and the relationship to the resolution of symptoms was recorded.

Primary data collection (medical records and interviews) was performed independently by two medical students at two time points after revisional surgery during the summer of 2015 and the fall of 2018. An additional assessment of the primary endpoint was performed between January 2019 and August 2020 for all patients to ensure that sufficient time had elapsed since the last surgery. Thus, the minimum follow-up was 2 years after the first surgical revision and 1 year after the last abdominal surgery. The medical students’ instructions emphasized that they should assess symptom development as objectively as possible.

The data were analyzed using descriptive statistics. This study was approved by the Swedish central ethical review board (Dnr Ö 17–2015).

## Results

**Fig. 1:**
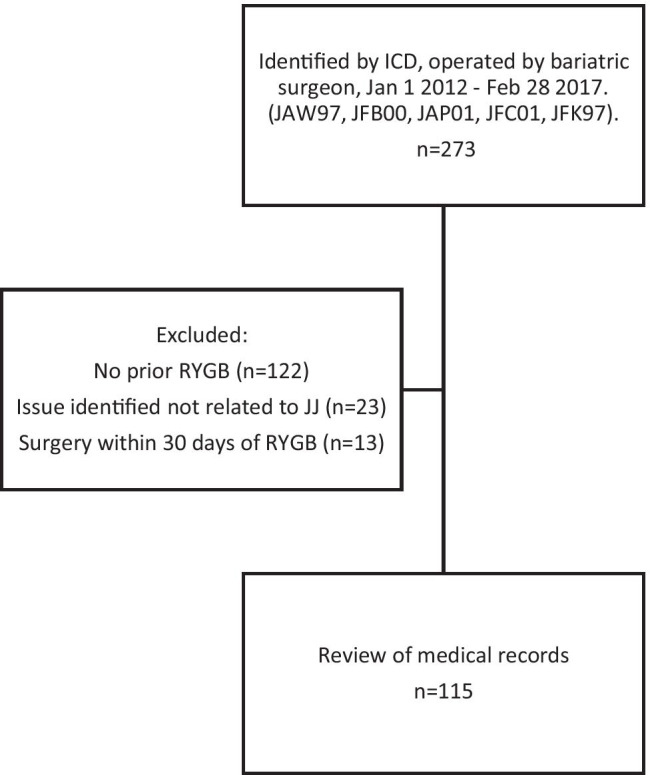
Flowchart of the inclusion of patients with a prior Roux-en-Y gastric bypass (RYGB) who underwent revisional surgery due to suspected dysfunction of the jejunojejunostomy (JJ)

A flowchart of patient inclusion is shown in Fig. 1. Patients who were excluded due to issues not related to the JJ had reconstruction of the JJ due to reasons other than JJ dysfunction, such as ischemic Roux limb after internal or ventral hernias, and reconstruction of the gastrojejunostomy followed by JJ reconstruction to avoid having too short of a Roux limb. The baseline characteristics of the study cohort at the time of primary RYGB are shown in Table 1 and compared to the baseline characteristics of participants in the national register for bariatric surgery (SOReg) from 2013–2017. The mean BMI at the time of follow-up was 29.1 kg/m^2^ (*n* = 85), which is similar to the mean BMI seen in SOReg (28.5 kg/m^2^ 2 years and 30.1 kg/m^2^ 5 years after RYGB).

**Table 1:** Descriptive statistics for patients at the time of Roux-en-Y gastric bypass compared to the Scandinavian Obesity Surgery Register (SOReg)

	Study group	SOReg (2013–2017)
Number of patients	115	23,802
Female	104 (90)	18,351 (77.1)
Body mass index, kg/m^2^	42.8 (21.3–79)	41.2
Patients with type 2 diabetes	15 (13)	(12.2)
Patients with pain disorder^a^	39 (34)	N/A
Musculoskeletal pain, %	N/A	16.3
Psychiatric disease (current or previous)	59 (51)	N/A
Depression (current)	N/A	3904 (16.4)
Psychiatric medication	39 (34)	N/A
Prior bariatric surgery (vertical banded gastroplasty or banding)	8 (7)	833 (3.5)
Smokers	29 (25)	3193 (13.9)
Both mesenteric defects closed at RYGB	45 (39)	N/A
All cause 30-day complications	25 (22)	1654 (7.2)^b^
Leak or obstruction at the jejunojejunostomy	8 (32)	N/A

Data are given as *n* (%) or mean (range) unless otherwise noted. *N/A*, not available

^a^Diagnosis of either chronic pain or a disease known to have chronic pain, such as rheumatoid arthritis

^b^Patients with prior bariatric surgery not included (*n* = 22,969)

Both mesenteric defects (Petersen’s space and at the JJ) were closed at the time of RYGB in 39% of patients, and only the defect at the JJ was closed in 4% of patients. Data on mesenteric defect closure were missing in 18% of patients.

Before the revision, patients could often tolerate fluids, whereas solid food caused postprandial pain, sometimes together with postprandial hypoglycemia. The pain was a cardinal symptom, and 109 (95%) patients reported pain as their main problem. Six (5%) patients did not report postprandial pain. Most patients reported postprandial pain localized to the upper left quadrant of the abdomen. Postprandial nausea was also common (71%), but retching and vomiting were reported less frequently (43%). Twenty-six percent of patients reported substantial problems with symptoms of postprandial hypoglycemia, whereas 73% reported none or only minor postprandial hypoglycemia. The median time from RYGB to symptom onset was 18 months (range 0–169 months).

**Fig. 2:**
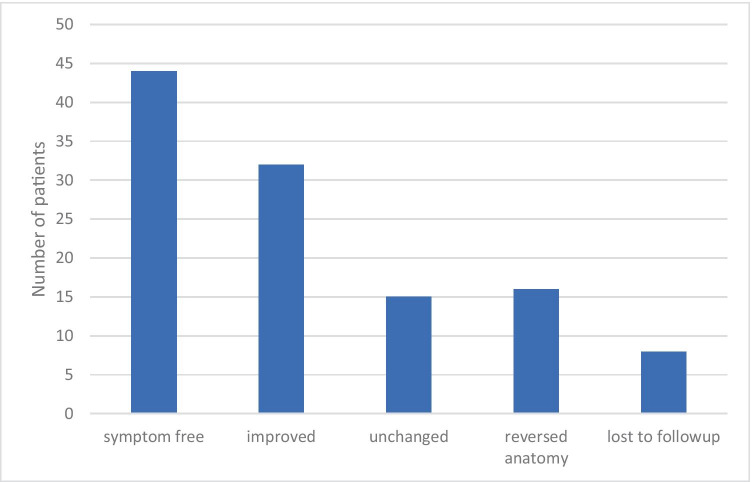
Subjective change in symptoms after laparoscopic surgery with revision of the jejunojejunostomy (JJ) after Roux-en-Y gastric bypass surgery. Symptoms that were monitored were abdominal pain, nausea, and/or hypoglycemia > 30 days after the primary procedure

Thirty-eight percent of our patients experienced complete and lasting resolution of symptoms following revisional surgery (Fig. 2). Another 28% experienced substantial and lasting improvement but were not entirely free from symptoms. Thirteen percent experienced no lasting improvement, and 14% underwent a reversal of the gastric bypass due to persisting symptoms. Seven percent were lost to follow-up.

Although more women than men undergo bariatric surgery, there were proportionally fewer men than expected in the study cohort (10% vs. 22.9% having bariatric surgery during the study period, Table 1). Regarding outcomes, 6 (55%) men were completely symptom-free at follow-up, 1 (9%) was improved, 1 (9%) had no improvement, and 2 (18%) underwent RYGB reversal.

Resolution of symptoms in the 8 patients (7%) with bariatric surgery prior to the RYGB demonstrated similar outcomes as the entire cohort, with 50% symptom-free, 40% improved, and 10% lost to follow-up.

Also, among the 39 patients with a chronic pain disorder at the time of RYGB, the results were similar to the entire cohort. At follow-up, 36% were symptom-free, 23% improved, 13% were not improved, and 20% had had RYGB reversal. Three patients (8%) were lost to follow-up.

Thirty-one patients (27%) were free from symptoms after primary RYGB until having emergency surgery due to internal herniation. These patients had emergency surgery with repositioning of the herniated small bowel and closure of the mesenteric defects, after which symptoms of a dysfunctional JJ arose and subsequently led to revisional surgery of the JJ.

An analysis of the number of abdominal surgeries after RYGB (including internal herniations and diagnostic laparoscopies at other hospitals) revealed that up to two or three laparoscopies were associated with symptom improvement, whereas additional surgeries seldom led to symptom relief. At the time of the revisional surgery, the laparoscopy often revealed kinking at the JJ. A frequent finding was that the JJ was adherent to the proximal part of the left side of the Roux limb. This left the JJ in a high position in the abdomen and, thus, prone to kinking due to the caudal traction of a filled Roux limb.

Surgical correction aimed at restoring the mobility of the JJ by adhesiolysis and often through opening and re-closing the mesenteric defect at a better angle to allow more mobility. We were increasingly prone to dividing the mesentery up to 10 cm between the gastrojejunostomy and JJ to allow the anastomosis to descend into a more caudal position and, hypothetically, prevent re-adhesion of the JJ to the Roux limb, and thus, decreasing the risk of JJ angulation. When the overall function of the JJ was in doubt, especially if previous surgeries with adhesiolysis had been performed, a low threshold was applied to resect and reconstruct the JJ by either laparoscopy or mini-laparotomy. Over time, and with increased experience, a more definitive surgical approach was applied to permanently resolve problems.

Our current clinical algorithm is to perform adhesiolysis and prolonged mesenteric division and most often renewed closure of mesenteric defects. If the patient experiences reoccurring symptoms, the next step will be to resect and reconstruct the JJ. For patients with severe eating/nutritional problems and/or unresolved complex hypoglycemia, reversal of RYGB may be indicated.

Follow-up of patients undergoing reversal of anatomy was not within the scope of this study, but will be investigated in a separate project.

We found no mortality or intensive care unit treatment associated with the reoperations. Although complications from revisional surgery were not formally assessed in this study, few were generally noted.

## Discussion

RYGB is an effective bariatric procedure with low complication rates overall, but late complications associated with internal herniation and abdominal pain are a concern [2]. In this study, we characterized a subgroup of patients who underwent RYGB and presented to a tertiary referral center with postprandial problems (i.e., pain, nausea, and/or complex hypoglycemia) leading to remedial laparoscopic intervention, for suspected dysfunction of the JJ. Remedial surgery resulted in symptom improvement or resolution in two-thirds of the patients.

The true prevalence of JJ dysfunction after RYGB is difficult to determine, especially as patients in this study underwent RYGB over a long time span and in many clinical settings. Furthermore, diagnostic criteria are not yet available for this phenomenon. A perceived increase in the incidence after 2008 coincides with a steep increase in the number of bariatric procedures and the establishment of new clinics in Sweden, which could be expected due to learning curves for both the surgeons and sites. In addition, many patients suffering from symptoms suggestive of dysfunction (postprandial pain and nausea) may have been considered to have poor compliance with dietary instructions or “dumping.” Such a misinterpretation may result in the phenomenon being underdiagnosed.

There has been an increasing trend of mesenteric defect closure since 2010 that parallels a registry-based randomized trial in Sweden. Stenberg et al. showed that closing mesentery defects increased the risk of remedial surgery due to bowel obstruction over the first year after surgery, even though the risk of internal herniations was reduced by 50% after 3 years [6]. The interpretation of these data is that closure of the mesenteric defects is important for avoiding internal herniation in the long term but can add a novel risk for early bowel obstruction. These clinical observations were one of the reasons we initiated the present study. Therefore, complications related to the JJ may, to a substantial extent, be secondary to the effects of mesenteric defect closure and that a learning curve may have increased the number of patients with JJ problems in Sweden. Our study also demonstrated that symptoms of JJ dysfunction can arise after emergency surgery for internal hernia, further suggesting that closure of the mesenteric defects constitutes a risk factor.

A comprehensive French study demonstrated that the risk of abdominal pain and late adverse events is relatively low after RYGB but substantially greater than in the background population [15]. A previous analysis in Sweden (unpublished data from SOReg) showed that a history of previous bariatric surgery (i.e., revisional surgery) is a risk factor for subsequent complications after RYGB [16]. Pierik et al. reported a higher risk of chronic abdominal pain after RYGB among patients with a previous bariatric procedure [4]. That 7% of our cohort was patients with previous bariatric procedures suggests that revision of previous bariatric surgery to RYGB increases the risk of subsequent JJ dysfunction, possibly due to increased complexity of the RYGB procedure and/or increased risk of adhesions. However, remedial surgery in this group appears to be as beneficial as after primary RYGB.

Chahal-Kummen et al. reported that pre-existing chronic abdominal pain is a risk factor for chronic abdominal pain 2 years after RYGB [17]. Notably, other outcomes (e.g., weight loss, resolution of comorbidity) were similar for patients with pre-existing chronic pain disorders and patients without these disorders. Patients with pre-existing chronic pain disorders prior to RYGB did not appear to have less beneficial outcomes after revisional surgery in our study.

The 30-day complication rate at the time of primary surgery was higher in our cohort than the mean for all RYGB procedures registered in SOReg during the same period. This suggests that unsuccessfully resolved problems or the effects of complications may increase susceptibility to late complications. In a 5-year follow-up study assessing chronic abdominal pain after RYGB, Hogestøl et al. demonstrated that early complications/reoperations are associated with a trend towards more chronic abdominal pain and repeat abdominal surgeries [18].

The possibility of an association between postprandial hypoglycemia and JJ dysfunction arose during the study period and was not the primary focus of this study which may leave hypoglycemic symptoms underdiagnosed in our data. However, a previous study suggested an association between severe postprandial hypoglycemia and dysfunction/partial obstruction at the JJ [5].

The weaknesses of this study include the retrospective and cross-sectional design, which make causal factors uncertain. The strengths of this study include a relatively large patient group with symptoms that have not been systematically addressed previously and a relatively long follow-up period. Although we obtained a clearer understanding and definition of the problem, factors predicting which patients that will improve with surgery remain to be identified, as well as the recommended type of surgical revision. Such factors should be studied prospectively.

## Conclusions

Surgically correctable problems with dysfunction at the JJ appear to be relatively common in patients with postprandial pain and nausea, and even complex hypoglycemia symptoms, after RYGB. Most patients with symptoms suggestive of JJ dysfunction experience improvement or total relief following revisional surgery. However, a substantial minority have persistent problems, with one in five eventually undergoing reversal of the anatomy. Future studies should address how to prevent JJ dysfunction during primary surgery and prospectively evaluate different strategies of surgical revision.
